# The association between blood pressure control and long-term cardiovascular outcomes in Hypertension coexistent with obstructive sleep apnea

**DOI:** 10.1186/s12872-023-03595-z

**Published:** 2023-11-21

**Authors:** Xiaoguang Yao, Nanfang Li, Mulalibieke Heizhati, Yingchun Wang, Yue Ma, Run Wang, Delian Zhang, Qin Luo, Junli Hu, Menghui Wang, Qing Zhu

**Affiliations:** 1 Hypertension Center of People’s Hospital of Xinjiang Uygur Autonomous Region, NO. 91 Tianchi Road, 830001 Urumqi, Xinjiang, China; 2 National Health Committee Key Laboratory of Hypertension Clinical Research, Urumqi, Xinjiang, China; 3 Xinjiang Hypertension Institute, Urumqi, Xinjiang China; 4 Key Laboratory of Xinjiang Uygur Autonomous Region “Hypertension Research Laboratory”, Urumqi, China

**Keywords:** Obstructive sleep apnea, Diastolic blood pressure, Major adverse cardiovascular and cerebrovascular event, Stroke

## Abstract

**Purpose:**

The goal of blood pressure (BP) control will be lower when hypertensive patients have comorbidities that can affect the risk of cardiovascular diseases. But, the goal of BP control for hypertensive patients coexistent with obstructive sleep apnea (OSA) is not discussed, which is a special population at high risk of cardiovascular diseases.

**Patients and methods:**

Using data from a retrospective study(Urumqi Research on Sleep Apnea and Hypertension (UROSAH) study, we enrolled 3267 participants who were diagnosed with hypertension and performed polysomnography during 2011–2013 to explore the association between BP control and long-term major adverse cardiovascular and cerebrovascular event (MACCE). Outcomes of interest was the levels of BP control, MACCE, cardiac event and cerebrovascular event. Then we calculated the cumulative incidence of MACCE and performed Cox proportional hazards with stepwise models.

**Results:**

379 of 3267 patients experienced MACCE during a median follow-up of 7.0 years. After full risk adjustment, BP control of 120-139/80-89mmHg was associated with the lowest risk of cerebrovascular event (HR: 0.53, 95%CI:0.35–0.82) rather than MACCE and cardiac event in the total cohort. The association did not change much in patients with OSA. When the SBP and DBP were discussed separately, the SBP control of 120-139mmHg or < 120mmHg was associated with the decreased incidence of MACCE and cerebrovascular event. When DBP control < 80 mm Hg, the risk of cerebrovascular event showed 54% decrease [(HR:0.46, 95%CI: 0.25–0.88)] in patients with hypertension and OSA.

**Conclusion:**

In this retrospective study, antihypertensive-drug-induced office and home BP control at 120-139/80-89mmHg showed possible beneficial effect on incident MACCE. However, current results need to be verified in future studies.

## Introduction

Obstructive sleep apnea (OSA) is a disorder characterized by recurrent episodes of upper airway obstruction during sleep, resulting in chronic intermittent hypoxia and sleep fragmentation. OSA is widely accepted as one of the most important causes of secondary hypertension, even though the hypertension may not necessarily be a consequence of the OSA. About 30–50% of hypertensive patients will have comorbid OSA [1]. Moreover, there is evident that the blood pressure (BP) can only be mildly decreased (3mmHg) after treatment with continuous positive airway pressure (CPAP) in OSA patients coexistent with hypertension [2]. Besides of hypertension, OSA is a common cardiovascular risk factor and related to coronary heart disease, heart failure, stroke, and et al. [3]. Nowadays, there are more than 1.2 billion patients with hypertension and nearly 1 billion patients with OSA globally [4]. It can be seen that both OSA and hypertension are not only common conditions, but often coexist. Thus, the individuals with hypertension and OSA are the special population that needs more attention, considering that both are known risk factors for cardiovascular and cerebrovascular diseases.

The pathophysiological changes caused by OSA, such as the intermittent hypoxia, hypercoagulable state [5], excessive mechanical stress on the heart and large artery walls caused by strong intrathoracic pressure changes, and the repetitive BP rise by arousal-induced reflex sympathetic activation [6] may greatly affect the perfusion pressure of coronary artery and cerebrovascular. Therefore, it is suspected that untreated OSA patients may need stricter BP control to have cardiovascular protection. However, there is no relevant evidence for this currently.

It is known that the antihypertensive goals are lower when hypertensive patients have comorbidities that can affect cardiovascular risk and treatment strategies [7]. However, this principle has been less verified in hypertensive patients with OSA. Moreover, a previous 8-week RCT has shown that valsartan induced a four-fold decrease in mean 24-hour BP than CPAP did in untreated hypertensive patients with OSA [8], suggesting that antihypertensive drugs are more helpful for OSA patients with hypertension to control BP, but the long-term benefit of BP control for CVDs outcomes in OSA has not been evaluated. Therefore, the aim of this study was to explore the possible goal of BP control via evaluating the association between BP control and the long-term cardiovascular consequences of the coexistence of hypertension and OSA.

## Materials and methods

### Study design and subjects

All subjects are from Urumqi Research on Sleep Apnea and Hypertension (UROSAH) study, which is a single-center retrospective cohort study. The inclusion and exclusion criteria have been described in our previous study [9]. At baseline, all subjects completed medical history collection and had height, weight, BP measurement, and overnight polysomnography (PSG) test. This study was conducted according to the Declaration of Helsinki and approved by the Ethics Committee of the People’s Hospital of Xinjiang Uygur Autonomous Region. Individual consent for this retrospective analysis was waived because all data were retrospectively collected and individual information was not disclosed.

### Diagnosis criteria

Hypertension is defined as SBP ≥ 140 mm Hg and/or DBP ≥ 90 mm Hg on 3 different days, or the current usage of any antihypertensive medication. OSA is defined as apnea-hypopnea index (AHI) ≥ 5 based on the in-laboratory polysomnography. Diabetes is defined as fasting glucose ≥ 7.0 mmol/L, the usage of glucose-lowering medication, or self-reported history of diabetes.

### Follow-up and outcomes

All participants were followed up through outpatient visits, inpatient medical records review, and telephone interviews by the trained nurses and physicians. The follow-up period ended Jan 2021, or first onset of major adverse cardiovascular and cerebrovascular event (MACCE) after the enrollment, or lost to follow-up.

For the participants who come to our hospital for follow-up used electronic sphygmomanometers (OMRON ABP-9021, Japan; Prodoctor BP3AJ1-1R, China) to measure upper arm BP in sitting positions in accordance with standard measurement procedures, all the BP measurement was performed by the trained nurses of our center during follow-up. The daytime BP readings at least 3 times on different days during the follow-up period were collected from the electronic medical record system of our hospital for the final analysis. For the patients who did not undergo follow-up visits at our hospital, the follow-up BP readings were obtained by the telephone interview. The readings of home BP monitoring were recorded as accurately as possible (at least 3 BP readings on the different days) or the range of BP monitoring in the recent 6 months were recorded, and confirmed the correct method of BP measurement. Finally, we also obtained BP monitoring information from participants who had regular annual BP measurement in their local hospitals. For patients who suffered MACCE, the level of BP before MACCE was collected via hospitalized medical records. After fully collecting the BP control levels of the participants during the follow-up period, we divided them into the following three groups according to their BP control levels: ≥140/90mmHg, 120–139/80-89mmHg and < 120/80mmHg.

As well, the treatment of OSA was asked, especially for the use of CPAP was recorded. Regular use of CPAP was defined as average use ≥ 4 h/night for > 70% of the follow-up period. The others with no or less than regular use were thought as ‘untreated’. For the patients whose BP controls ≥ 140/90mmHg or “untreated OSA” during follow-up were suggested to come to our center or the local hospital for further consultation and adjusted the antihypertensive treatment.

**Fig. 1:**
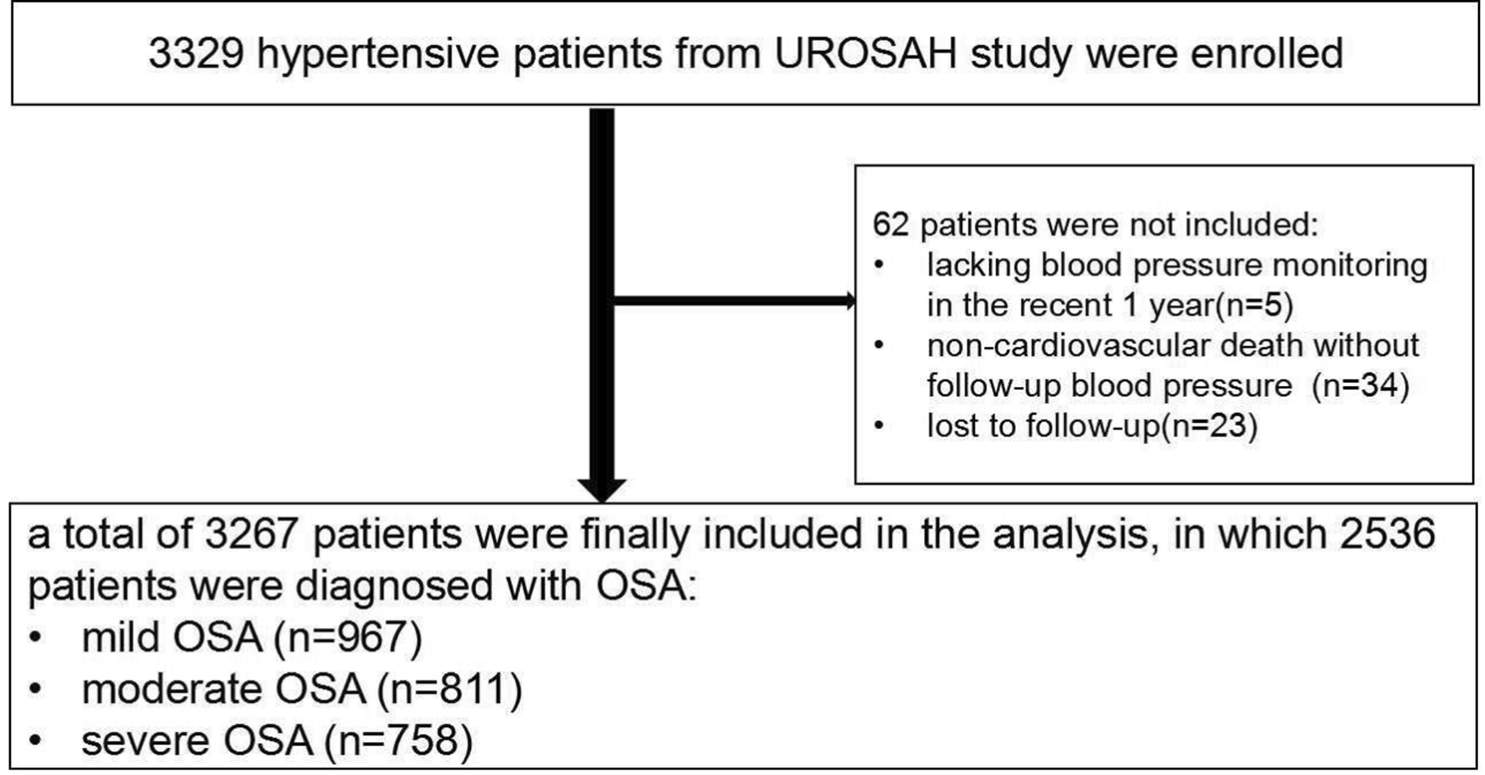
Flowchart of the study. UROSAH: Urumqi Research on Sleep Apnea and Hypertension; OSA:obstructive sleep apnea; CVDs: cardiovascular diseases

The primary endpoints were the first onset MACCE after the enrollment during the follow-up period, including fatal and non-fatal cardiovascular event and cerebrovascular event. All endpoints were defined in accordance with the proposed definitions by the Standardized Data Collection for Cardiovascular Trials Initiative [10]. In the present study, cardiovascular event included fatal or non-fatal myocardial infarction (MI), revascularization (percutaneous coronary intervention and coronary artery bypass grafting), and cardiac rehospitalization due to unstable angina or heart failure. Cerebrovascular event included fatal and non-fatal strokes (ischemic stroke, hemorrhagic stroke, and TIA). If the patient was diagnosed with MACCE in our hospital, the results of the evaluation were recorded in the electronic medical record system. If MACCE was found and diagnosed beyond our hospital, the time when the endpoint event occurred and the hospital where it was diagnosed and treated was asked, then the patients were asked to provide the diagnosis and treatment data. For the sudden death, the cause of death was asked from bereaved relatives and verified by the hospital death certificate or hospitalization data, or the local police substation. All the clinical events were confirmed by medical documents and identified by the clinical event committee of our hospital. The international classification of diseases (ICD-10) classification code was used to classify cases of a deadly disease. In the study, the first events were collected for the final analysis. The detailed number of endpoints was as follows: fatal AMI(n = 16), fatal stroke (n = 6), nonfatal AMI(n = 204), nonfatal stroke(n = 132), TIA(n = 1), cardiac revascularization (n = 102) and non-cardiovascular death(n = 20). Finally, 3267 patients with complete follow-up information were included for analysis in this study (Fig. 1).

### Statistical analysis

Continuous variables were reported as mean ± standard deviation (SD) if normally distributed and as the median and interquartile range (IQR) if not. Differences between the two groups for normally distributed continuous variables were compared using the independent samples t-test and the Mann Whitney U test were used for non-normally distributed continuous variables. Categorical variables were presented as observed numbers and percentages and compared using the Pearson Chi-square test among groups. The cumulative incidence of primary outcomes was estimated by Kaplan-Meier survival curves, and the log-rank test was used to estimate the difference between the different levels of BP control. Cox proportional hazards models were performed to test the association between CVDs outcomes (including MACCE, cardiac event, and cerebrovascular event) and the levels of BP control in the total cohort and patients with OSA. The risk factors such as age, sex, body mass index, baseline systolic BP and diastolic BP, AHI, low-density lipoprotein cholesterol, eGFR, smoking, type 2 diabetes, and the usage of drugs were adjusted. Furthermore, to eliminate the impact of OSA-specific treatment on the association between BP control and MACCE, we conducted the sensitivity analysis in the OSA population by excluding individuals who use CPAP regularly. Data were analyzed using SPSS statistical software (version 25.0, SPSS Inc., Chicago, Illinois). All analyses were two-tailed and a P value of < 0.05 was statistically significant.

## Results

### Baseline characteristics of the participants

A total of 3267 patients (2138 males and 1129 females) was included and divided into 3 groups by the follow-up BP. Table 1 showed the baseline characteristics of the total cohort and the participants among 3 groups. The mean age was 48.6 years and 77.6% patients were diagnosed as OSA at baseline. 379 out of 3267patients experienced MACCE during a median follow-up of 7.0 years.

**Table 1:** Baseline and follow-up characteristics of the patients by the levels of blood pressure control

	Total cohort (n = 3267)	≥ 140/90mmHg (n = 1789)	120–139/80-89mmHg (n = 993)	< 120/80mmHg (n = 485)	P value
Baseline anthropometric indices					
Age (yr)	48.6 ± 10.9	47.9 ± 10.9	49.3 ± 10.8	49.4 ± 11.0	0.002
Gender (Male,%)	2138(65.4)	1198(67.0)	649(65.4)	291(60.0)	0.017
BMI (kg/m^2^)	28.0 ± 3.8	28.3 ± 3.9	27.8 ± 3.6	27.2 ± 3.7	< 0.001
Baseline office SBP (mmHg)	139.6 ± 19.5	142.5 ± 19.7	136.9 ± 18.9	134.7 ± 18.2	< 0.001
Baseline office DBP (mmHg)	91.7 ± 13.9	93.7 ± 13.8	90.0 ± 13.9	88.2 ± 12.8	< 0.001
Smoker (n,%)	1071(32.8)	620(34.7)	317(31.9)	134(27.6)	0.011
Baseline biochemical tests					
GGT (mmol/L)	28.4 ± 22.2	29.0 ± 24.3	27.1 ± 19.1	28.8 ± 20.1	0.109
GOT (mmol/L)	22.2 ± 14.2	22.5 ± 17.0	21.4 ± 9.4	22.5 ± 10.1	0.119
eGFR (ml/min/1.73m^2^)	96.9 ± 21.8	97.5 ± 22.1	97.1 ± 21.6	94.6 ± 21.3	0.031
LDL-c (mmol/L)	2.6 ± 0.8	2.6 ± 0.8	2.6 ± 0.8	2.5 ± 0.8	0.068
Baseline PSG parameters					
Total sleep time (mins)	390.3 ± 51.8	390.6 ± 52.5	391.5 ± 52.7	386.7 ± 47.1	0.297
Sleep efficiency (%)	73.5 ± 9.6	73.6 ± 9.8	73.6 ± 9.3	72.8 ± 9.6	0.335
AHI (event/h)	14.2(5.6–26.8)	14.9(5.9–27.9)	18.5 ± 17.9	18.1 ± 17.8	0.003
Mean SaO_2_ (%)	92.4 ± 2.7	92.3 ± 2.7	92.4 ± 2.8	92.5 ± 2.5	0.241
Nadir SaO_2_ (%)	80.7 ± 7.6	80.3 ± 7.7	80.9 ± 7.7	81.7 ± 6.9	0.001
CHD (n,%)	361(11.0)	203(11.3)	106(10.7)	52(10.7)	0.837
Diabetes (n,%)	540(16.5)	307(17.2)	151(15.2)	82(16.9)	0.401
OSA (n,%)	2536(77.6)	1416(79.2)	756(76.1)	364(75.1)	0.063
Mild OSA (n,%)	967(29.6)	529(29.6)	304(30.6)	134(27.6)	
Moderate OSA (n,%)	811(24.8)	456(25.5)	223(22.5)	132(27.2)	
Severe OSA (n,%)	758(23.2)	431(24.1)	229(23.1)	98(20.2)	0.076
Antihypertensive regimen (n,%)					
0–1 drug	1160(35.5)	532(29.7)	421(42.4)	207(42.7)	< 0.001
2 drugs combination	1635(50.0)	959(53.6)	451(45.7)	222(45.8)	
≥3 drugs combination	472(14.4)	298(16.7)	118(11.9)	56(11.5)	
Lipid-modifying agents (n,%)	2030(62.1)	1135(63.4)	607(61.1)	288(59.4)	0.193
Antiplatelet drugs (n,%)	1601(49.0)	862(48.2)	493(49.6)	246(50.7)	0.544
Antidiabetic drugs use (n,%)	479(14.7)	262(14.6)	140(14.1)	77(15.9)	0.662
Follow-up mean SBP (mmHg)	136.7 ± 16.9	146.7 ± 15.8	129.3 ± 5.3	116.0 ± 5.9	< 0.001
Follow-up mean DBP (mmHg)	86.3 ± 12.1	92.9 ± 11.3	80.1 ± 6.2	74.5 ± 6.8	< 0.001
Follow-up, median (IQR),y	7.0(6.0-8.1)	7.0(6.1-8.0)	7.0(6.1–8.1)	6.9(5.8-8.0)	0.221
Person-years followed,y	22017.5	11894.0	6641.7	3097.3	
Total number of MACCE (n)	379	225	99	55	
Outcome per 1000 person-years	17.2	18.9	14.9	17.7	

BMI: body mass index, SBP: systolic blood pressure, DBP: diastolic blood pressure, GGT:Alanine transaminase, GOT: Aspartate transaminase, eGFR: glomerular filtration rate, LDL-C: low-density lipoprotein cholesterol, AHI: apnea hypopnea index, SaO_2_:saturation, CHD: coronary heart diseases; OSA: obstructive sleep apnea; The mean level of follow-up BP was calculated in 3267 patients

### MACCE incidence

**Fig. 2:**
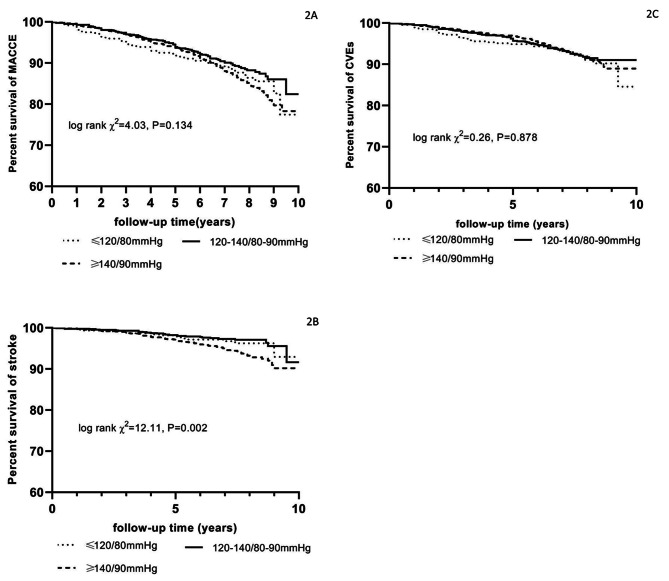
Kaplan-Meier curve of cardiovascular outccomes. Proportion survival of MACCE(2 A), stroke(2B), and CVEs(2 C) in different levels of blood pressure control. *MACCE: major adverse cardiovascular and cerebrovascular event; CVEs: cardiac event

Figure 2 illustrated the crude incidence of MACCE, cardiac event, and cerebrovascular event in different groups of BP control. The incidence of stroke decreased significantly when BP was not controlled < 140/90mmHg (log-rank test, P = 0.022, Fig. 2B). Furthermore, the incidence of stroke of pairwise comparison between the three groups was as follows: ≥140/90mmHg vs. 120–139/80-89mmHg (χ^2^ = 3.44, P = 0.064), ≥ 140/90mmHg vs. < 120/80mmHg (χ^2^ = 10.37, P = 0.001), and 120–139/80-89mmHg vs. < 120/80mmHg (χ^2^ = 10.36, P = 0.001). But no significant difference was observed between BP control and MACCE and cardiac event (Fig. 2A C).

### BP control and the incident MACCE in the total cohort

Table 2 presented the association between BP control and the incident MACCE, cerebrovascular event, and cardiac event in the total cohort. After full risk adjustment, BP control of 120–139/80-89mmHg was associated with the lowest risk of cerebrovascular event only(HR: 0.53, 95%CI: 0.35–0.82). Separately, the SBP control of 120-139mmHg was associated with the lowest HR of the incident MACCE and cerebrovascular event. Compared to patients with DBP ≥ 90mmHg, patients with BP controlled lower than 80mmHg had a reduced risk of cerebrovascular event by approximately 50% [(HR:0.51, 95%CI: 0.29–0.88)]. However, no benefits of SBP control and DBP control for cardiac event were observed.

**Table 2:** The association of blood pressure control and MACCE, cerebrovascular event and cardiac event in total cohort (n = 3267)

Total cohort	MACCE (n = 379)		cerebrovascular event (n = 138)		Cardiac event (n = 220)		
**BP control (mmHg)**	HR(95%CI)	P value	HR(95%CI)	P value	HR(95%CI)	P value	
≥ 140/90	1.0		1.0		1.0		
120–139/80–89	0.83(0.65–1.06)	0.129	0.53(0.35–0.82)	0.004	1.03(0.76–1.40)	0.860	
<120/80	1.10(0.81–1.48)	0.552	0.69(0.40–1.20)	0.192	1.31(0.88–1.93)	0.183	
**SBP control (mmHg)**	HR(95%CI)	P value	HR(95%CI)	P value	HR(95%CI)	P value	
≥ 140	1.0		1.0		1.0		
120–139	0.73(0.58–0.91)	0.006	0.46(0.31–0.69)	< 0.001	0.93(0.69–1.25)	0.624	
< 120	0.90(0.67–1.21)	0.480	0.58(0.34–0.97)	0.039	1.09(0.74–1.60)	0.660	
**DBP control (mmHg)**	HR(95%CI)	P value	HR(95%CI)	P value	HR(95%CI)	P value	
DBP ≥ 90	1.0		1.0		1.0		
85 ≤ DBP < 90	0.71(0.51–0.98)	0.040	0.72(0.43–1.20)	0.210	0.81(0.55–1.18)	0.270	
80 ≤ DBP < 85	0.84(0.64–1.09)	0.185	0.69(0.44–1.07)	0.100	0.93(0.66–1.31)	0.689	
DBP < 80	0.71(0.53–0.96)	0.026	0.51(0.29–0.88)	0.016	0.71(0.46–1.10)	0.123	

*HR: hazard ratio, BP: blood pressure. Adjusted for age, sex, body mass index, baseline systolic blood pressure and diastolic blood pressure, apnea hypopnea index, low-density lipoprotein cholesterol, eGFR, smoking, type 2 diabetes, lipid-lowering drugs, antidiabetic drugs and antiplatelet drugs

### BP control and the incident MACCE in patients with and without OSA

As to patients with OSA, compared with the subjects with SBP ≥ 140mmHg, the risk of cerebrovascular event was significantly reduced in patients with BP control < 140mmHg, and the HR was very similar in the 120-139mmHg and < 120mmHg groups. Taking DBP > 90mmHg as a reference, the risk of MACCE decreased by 29% when DBP < 80mmHg, while the risk of cerebrovascular event could decrease by 54%. However, it had not yet been observed that a decrease in BP can bring significant benefits from cardiac event. In the non OSA population, we observed a decrease in the risk of MACCE and cerebrovascular event when SBP control < 140mmHg or DBP control < 90mmHg, although there was no statistically significant difference (Table 3).

**Table 3:** The association between blood pressure control and incident MACCE, cerebrovascular event, and cardiac event in hypertensives with and without OSA

	Patients with OSA			Patients without OSA		
	MACCE (n = 318)	cerebrovascular event (n = 106)	Cardiac event (n = 194)	MACCE (n = 61)	cerebrovascular event (n = 32)	Cardiac event (n = 26)
BP control (mmHg)	HR(95%CI)	HR(95%CI)	HR(95%CI)	HR(95%CI)	HR(95%CI)	HR(95%CI)
≥ 140/90	1.0	1.0	1.0	1.0	1.0	1.0
120–139/80–89	0.89(0.69–1.15)	0.55(0.34–0.90)	1.07(0.77–1.47)	0.63(0.33–1.22)	0.51(0.20–1.29)	0.84(0.32–2.21)
< 120/80	0.99(0.71–1.41)	0.60(0.31–1.17)	1.18(0.77–1.82)	1.54(0.81–2.94)	0.97(0.36–2.64)	1.87(0.70–4.99)
SBP control (mmHg)						
≥ 140	1.0	1.0	1.0	1.0	1.0	1.0
120–139	0.78(0.61–0.99)	0.52(0.34–0.82)	0.93(0.68–1.27)	0.53(0.28–0.99)	0.30(0.12–0.76)	1.07(0.41–2.79)
< 120	0.83(0.60–1.16)	0.53(0.28–0.98)	0.99(0.66–1.52)	1.18(0.62–2.25)	0.72(0.28–1.84)	1.71(0.60–4.91)
DBP control (mmHg)						
DBP ≥ 90	1.0	1.0	1.0	1.0	1.0	1.0
85 ≤ DBP < 90	0.76(0.53–1.07)	0.71(0.40–1.27)	0.79(0.50–1.24)	0.52(0.20–1.38)	0.81(0.26–2.49)	0.24(0.03–1.86)
80 ≤ DBP < 85	0.83(0.62–1.11)	0.66(0.40–1.11)	0.90(0.62–1.31)	0.99(0.53–1.86)	0.84(0.34–2.06)	1.19(0.47–3.01)
DBP < 80	0.71(0.51–0.99)	0.46(0.25–0.88)	0.84(0.56–1.27)	0.76(0.36–1.63)	0.72(0.24–2.15)	0.56(0.17–1.86)

*HR: hazard ratio. BP:blood pressure, OSA: obstructive sleep apnea. For patients with OSA, age, sex, body mass index, baseline systolic blood pressure and diastolic blood pressure, low-density lipoprotein cholesterol, eGFR, apnea hypopnea index, smoking, type 2 diabetes, lipid-lowering drugs, antidiabetic drugs and antiplatelet drugs were adjusted. For patients without OSA, age, sex, baseline systolic blood pressure and diastolic blood pressure, smoking and type 2 diabetes were adjusted

### Sensitivity analysis

To clarify the effect of drug-induced BP control on MACCE in patients with OSA, sensitivity analysis was conducted by excluding 114 individuals who received regular CPAP treatment. Similar to the results in the total cohort, OSA patients with DBP < 80mmHg had a decrease in MACCE of about 29% [HR (95% CI): 0.71 (0.51–1.98), P = 0.040], OSA patients with DBP < 80mmHg had a decrease in MACCE of about 30% [HR (95% CI): 0.71 (0.51–1.98), P = 0.040], and a decrease in the risk of stroke of 58% [HR (95% CI): 0.42 (0.22–0.82), P = 0.011] taking DBP control ≥ 90mmHg as a reference, but no benefit from BP control was found for cardiac event, and BP < 120/80mmHg seemed to increase the risk of cardiac events (Table 4).

**Table 4:** Sensitivity analysis of the effect of blood pressure control on MACCE, cerebrovascular and cardiac event in OSA patients without CPAP treatment (n = 2422)

	MACCE (n = 303)		cerebrovascular event (n = 100)		Cardiac event (n = 185)	
**BP control(mm Hg)**	HR(95%CI)	P value	HR(95%CI)	P value	HR(95%CI)	P value
≥ 140/90	1.0		1.0		1.0	
120–139/80–89	0.85(0.65–1.11)	0.241	0.55(0.33 − 0.09)	0.018	1.01(0.72–1.40)	0.978
< 120/80	0.99(0.69–1.41)	0.939	0.50(0.24–1.05)	0.067	1.23(0.80–1.90)	0.345
Systolic BP(mhm)	HR(95%CI)	P value	HR(95%CI)	P value	HR(95%CI)	P value
≥ 140	1.0		1.0		1.0	
120–139	0.78(0.60-1.00)	0.052	0.54(0.34–0.85)	0.008	0.91(0.66–1.26)	0.559
< 120	0.85(0.60–1.19)	0.339	0.47(0.24–0.92)	0.028	1.06(0.69–1.61)	0.802
Diastolic BP(mmHg)	HR(95%CI)	P value	HR(95%CI)	P value	HR(95%CI)	P value
DBP ≥ 90	1.0		1.0		1.0	
85 ≤ DBP < 90	0.75(0.53–1.07)	0.112	0.72(0.40–1.28)	0.258	0.78(0.45–1.24)	0.289
80 ≤ DBP < 85	0.76(0.56–1.03)	0.080	0.57(0.33–0.99)	0.046	0.85(0.58–1.25)	0.403
DBP < 80	0.71(0.51–1.98)	0.040	0.42(0.22–0.82)	0.011	0.86(0.57–1.31)	0.490

*HR:hazard ratio. BP: blood pressure. 114 patients who received regular use of continuous positive airway pressure (CPAP) were excluded. Adjusted for age, sex, body mass index, baseline systolic blood pressure and diastolic blood pressure, low-density lipoprotein cholesterol, eGFR, apnoea apnoea hypopnea index, smoking, type 2 diabetes, lipid-lowering drugs, anti-diabetic drugs and anti-platelet drugs

## Discussion

It is mentioned in the latest 2023 ESH Guidelines for the management of arterial hypertension that all major classes of antihypertensive drugs can be used to reduce BP in patients with OSA, and CPAP application has small reductions in office BP values [2]. However, the goal of BP control in hypertension with OSA is currently unclear. In the present study, we observed antihypertensive-drug-induced office and home BP control at 120–139/80-89mmHg showed possible beneficial effect on incident MACCE in the total cohort after adjustment for risk confounders. Further, it seemed that there was an association between the benefits of cerebrovascular event and stricter BP control in patients with OSA. Our study may provide more novel information for BP management of hypertension and OSA.

2020 International Society of Hypertension(ISH) global hypertension practice guidelines suggested BP < 140/90mmHg as the essential target of office BP for treated hypertension and < 130/80mmHg as the optimal target for treated hypertensives aged < 65yrs [7], a lower target BP was strongly recommended also in some high CVDs risk groups [11]. In this study, when the systolic BP and diastolic BP were considered simultaneously, the lowest HRs of MACCE, cerebrovascular event and cardiovascular event were observed when BP was at 120–139/80-89mmHg compared to patients with BP control ≥ 140/90mmHg both in the total cohort and in patients with OSA, although P value did not reach to the statistical significance. However, due to a retrospective cohort study, recall bias of BP measurement was difficult to avoid, in the present study, for the patients who suffered MACCE, the following BP levels were based on the means of the BP measurements before MACCE, which would make the results more reliable and tend to underestimate the risk of the event.Our findings provided preliminary reference of the BP control for hypertension with OSA, especially for middle-aged hypertensives, who were thought to be a particularly important group in light of their uniquely elevated risk of CVDs morbidity and mortality [12].

When the systolic BP and diastolic BP control were considered separately, it was found that when SBP was controlled at 120-139mmHg, the risks of MACCE and cerebrovascular event in OSA patients with hypertension were reduced significantly. With the decrease of DBP, the risk of MACCE and cerebrovascular event significantly decreased, especially when the DBP < 80mmHg, the risk of cerebrovascular event can be reduced by about 50%. Similar to the results of ALLHAT study [13], our findings also supported that the risk pattern of SBP and DBP may differ by clinical outcomes in hypertension with OSA. As Franz Messerli wrote in his editorial, “the lower the better” is correct for stroke prevention. If there was no risk to the heart, the SBP with the best brain protection is 110–120 mmHg [14]. As well, the latest study found that intensive SBP control (SBP < 120mmHg) was associated with increased cerebral perfusion, most notably in participants with a history of CVDs [15]. However, the role of DBP in the prediction of CVDs is inconsistent. In 2017, the ACC/AHA hypertension-management guidelines did not consider DBP in the determination of cardiovascular risk [16]. But a very recent study pointed out that systolic and diastolic hypertension independently predicted adverse outcomes, no matter which threshold defines hypertension [17].

We did not analyze the association between BP control and cardiovascular mortality due to few dead cases. All subjects in our study were prescribed individualized antihypertensive regimens after systemic evaluation of hypertension, and if necessary, lipid-lowering drugs, anti-diabetic drugs and/or anti-platelet drugs were also used, which may attenuate the association between BP control and incident risk of MACCE. Moreover, as Diastolic Chronic Heart Failure Study (DIAST-CHF) explained, OSA did not show a significant adverse effect on cardiovascular morbidity and mortality in patients with cardiovascular risk factors [18], in which the effective pharmacological interventions and the limited number of severe OSA may be the possible reasons.

There are several limitations. Firstly, as the treatment rate of OSA was far from satisfactory [3, 19], and the SAVE trial even concluded that the use of CPAP did not prevent CVDs in patients with moderate-to-severe OSA and established CVDs [20], the effect of the combination of drug-induced BP control and CPAP treatment on MACCE was not assessed. However, these patients were taken antihypertensive drugs and followed up in our center, they provided information of the natural history of untreated OSA in hypertension. Secondly, although we used multiple methods to collect follow-up BP values of participants, most of them provided their office and home BP values rather than the recent report of the 24-hour ambulatory blood pressure monitoring, which may affect the assessment of BP level during follow-up. Thirdly, it is important to point out that this is a retrospective study, and the biggest drawback is the information of BP control levels during follow-up, especially for patients who had MACCE. Although we have tried to collect BP values from medical records before the occurrence of MACCE, information bias cannot be avoided. Our results require more rigorous prospective studies.

## Conclusion

In this retrospective study, antihypertensive-drug-induced BP control at 120–139/80-89mmHg showed possible beneficial effect on incident MACCE. However, current results need to be verified in future studies.

## Data Availability

The datasets used and/or analyzed during the current study are available from the corresponding author Prof. Nanfang Li on reasonable request.
